# Dr. Jared M. Newell

**Published:** 1903-07

**Authors:** 


Dr. Jared M. Newell, of Rochester, died April 23, 1903, aged 82 years. He graduated at the University of Buffalo in 1846.

